# Supplementary material for: Super-sectioning with multi-sheet reversible saturable optical fluorescence transitions (RESOLFT) microscopy
Source: Nat Methods. 2024 Feb 23;21(5):882–8. doi: 10.1038/s41592-024-02196-8 (PMC11093742; doi:10.1038/s41592-024-02196-8)
Supplement: Supplementary file 1 — Supplementary Notes 1–4, Supplementary Table 1, Supplementary Figs. 1–5 [file 41592_2024_2196_MOESM1_ESM.pdf]

nature methods

Article

https://doi.org/10.1038/s41592-024-02196-8

Super-sectioning with multi-sheet reversible saturable optical fluorescence transitions (RESOLFT) microscopy

In the format provided by the authors and unedited

Table of Contents

Supplementary Notes.......... 1
Supplementary Note 1: RSFP properties and performance.......... 1
Speed and fatigue of rsEGFP2 and rsEGFP(N205S).......... 1
Supplementary Note 2: Confinement estimation.......... 2
Supplementary Note 3: Simulations.......... 3
Sample and illumination.......... 3
Optical imaging.......... 3
Detection.......... 4
Supplementary Note 4: Optical build and alignment.......... 5
Primary objective and tube lens.......... 5
Galvanometric scanning unit.......... 5
Oblique optical detection.......... 5
Illumination paths.......... 6
Alignment module for tilt and translation.......... 7
Supplementary Tables.......... 8
Supplementary Table 1: Imaging parameters.......... 8
Supplementary Figures.......... 9
Supplementary Figure 1.......... 9
Supplementary Figure 2.......... 10
Supplementary Figure 3.......... 11
Supplementary Figure 4.......... 12
Supplementary Figure 5.......... 13
References.......... 14

Supplementary Notes

Supplementary Note 1: RSFP properties and performance

The presented Multi-sheet RESOLFT technology is, like previously described RESOLFT-based imaging techniques, based on the use of reversibly switchable fluorescent proteins (RSFPs). The light-induced reversible photo-switching and the switching states lifetime enable us to imprint spatial patterns of state distributions by illuminating the sample with pulse schemes of spatially structured light patterns. The performance of these systems is thus tightly linked to the on-off switching kinetics of the RSFP labels.

Most RESOLFT-based techniques demonstrated to date use so-called negative switchers. This means that the excitation and off-switching processes are spectrally coupled, meaning that they are induced by the same wavelength. For the RSFPs used in the presented data (rsEGFP2 and rsEGFP(N205S)), this wavelength is around 488 nm. For switching on the RSFPs, a wavelength of 405 nm is used.

Speed and fatigue of rsEGFP2 and rsEGFP(N205S)

rsEGFP2 is one of the fastest switchers of existing RSFPs, specifically regarding the switching from the on to the off-state. This allows for faster imaging since the on-to-off transition is often a rate-limiting step in the imaging sequence. However, due to the spectral coupling of negative switchers, the fast off-switching also means that the proteins will switch off quickly during the excitation step and on average give fewer fluorescent photons compared to a slower switcher. The other RSFP used in this work is the rsEGFP(N205S), which represents a slower switching RSFP in the green part of the spectrum. As described, labelling with rsEGFP(N205S) forces a slower imaging speed, but gives more photon emissions from each on-state fluorophore during an on-state cycle. Supplementary Fig. 1 a-b shows a comparison of the off-switching times of rsEGFP2 and rsEGFP(N205S) for different intensities of 488 nm off-switching illumination.

Another important property of the RSFPs for high-quality imaging, especially in time-lapse recordings, is resistance to switching fatigue. This refers to the proteins' ability to withstand prolonged illuminations and switching without losing their fluorescence or switching properties. Measurements show that strong fatigue resistance is tightly linked to switching speed in the sense that RSFPs that exhibit fast off-switching often also exhibit strong resistance to fatigue. Supplementary Fig. 1 c-d shows the fatigue resistance of rsEGFP2 and rsEGFP(N205S) for different intensities of 488 nm off-switching illumination. The graphs show an initial steep drop in fluorescence in the first 1-2 cycles. This phenomenon is attributed to the fact that the initial thermal equilibrium population can not be fully replenished using only photoinduced switching[1]. The subsequent loss of fluorescence is attributed to switching fatigue. It is clear from the graphs that rsEGFP2 shows significantly greater fatigue resistance. Apart from the clear difference between the two RSFPs, it is also apparent that, mainly for rsEGFP(N205S), the rate of fatigue increases as the intensity of the off-switching illumination increases, even though the total dose of 488 nm illumination is adjusted to switch off 80% of the RSFP population in every cycle. This further supports our belief that the low illumination doses used in Multi-sheet RESOLFT may provide additional benefits for long-term time-lapse imaging.

Supplementary Note 2: Confinement estimation

For RSFPs, we define the switching wavelengths as the wavelengths that efficiently induce photoswitching between the different states. In the off-state, the RSFP has a high absorption cross-section for the on-switching light. If absorption occurs, there is a high probability that the protein undergoes the conformational change associated with the switch from the off to the on state. Previous studies[2] have characterized the rate of photoswitching induced by the different wavelengths. At low illumination intensities (W-kW/cm^2), the rates of photoswitching can be assumed approximately proportional to the illumination intensity and can be expressed as

$$r_{on} = I * cs_{on}$$

$$r_{off} = I * cs_{off}$$

where r_{on} and r_{off} are the on and off-switching rates, I is the illumination intensity and cs_{on} and cs_{off} are the respective cross sections for the off-to-on and on-to-off transitions. For a given illumination intensity and time t, the probability of an on-state RSFP still being in the on-state at time t is then

$$p_{on}(t) = p_{on,\, t=0} * e^{-t*r_{off}}$$

where $p_{on,t=0}$ is the probability of the RSFP being in the on-state at $t = 0$. If an illumination induces both on and off-switching, the probability expression becomes

$$p_{on}(t) = \frac{r_{on}}{r_{on}+r_{off}} + \left(p_{on,\ t=0} - \frac{r_{on}}{r_{on}+r_{off}}\right) e^{-t*\left(r_{on}+r_{off}\right)}.$$

Using this equation together with a spatial intensity profile of the off-switching illumination, we can predict and plot how the population of on-state RSFPs is confined to the region around the zero-intensity point of the off-switching illumination as shown in Fig. 1b, left graph. By multiplying the on-state distribution by the intensity distribution of the read-out illumination the expected emission profile can be estimated. The FWHM of the expected emission profile is plotted in the right graph of Fig. 1b for increasing illumination time with the off-switching light. For the graphs shown in Fig 1b, switching properties of the RSFP are set to mimic the rsEGFP2 protein with cs_{off} = 7.15 (units is rate/(kW/cm^2) where the rate is defined as events/millisecond) and a maximum illumination intensity of 50 W/cm^2.

Supplementary Note 3: Simulations

For the simulations performed and shown in Fig. 1d, a simulation tool developed in-house was used to generate the virtual data. The data tool can be conceptually divided into several three main parts.

Sample and illumination

The first part simulates the physics in sample space and incorporates the geometry of the sample, the behavior of the labels and the interaction with the illuminations. The sample geometry is defined by an array of 3D coordinates, representing the positions of individual fluorophores. These coordinates are constant throughout the simulations. When simulating RSFP labelled samples, the fluorophore properties are set by assigning each fluorophore type a set of parameters describing a property called the cross-section for the on, the off and the emission respectively. The cross sections for on and off-switching are defined as in Supplementary Note 2 and the cross section for fluorescence emission is defined as

$$r_{em} = I * cs_{em}$$

Where r_{em} is the rate of fluorescence emission if the RSFP is residing in the on-state. If the RSFP is residing in the off-state, no fluorescence emission is elicited. The amount of emission is also accompanied by an emission spectrum, which allows for applying detection filters in later parts of the simulation.
The illumination patterns are defined using analytical functions (often sums of complex wave functions). When an illumination pulse is applied to the sample, the intensity at each fluorophore coordinate is calculated. Using these intensities and the time of illumination, a numerical simulation is performed for each fluorophore that generates a switching trace throughout the given illumination time using randomly generated on and off-state lifetimes. From each switching trajectory, the total time spent in the on-state can be extracted and the expected fluorescence emission can be calculated as

$$E[em] = r_{rm} * t_{on}$$

Where $E[em]$ is the expected number of emitted photons and t_{on} is the time spent in the on-state.
For the simulation shown in Fig. 1d, the label parameters were set to mimic the behaviour of the RSFP rsEGFP2. For this, the cross-sectional values for 405 nm and 488 nm illumination were set to (units of rate/(kW/cm^2) where rate is defined as events/millisecond)

$$405\,nm\!: cs_{on} = 5,\ cs_{off} = 1.24,\ cs_{em} = 12$$
$$488\,nm\!: cs_{on} = 0.3,\ cs_{off} = 7.15,\ cs_{em} = 68.5$$

Optical imaging

When an expected emission has been generated from each fluorophore, the optical imaging of the sample is simulated by first transforming the emission information from each fluorophore into a voxelized volume representing the expected total emission from each voxelized point in 3D space. Then a 3D convolution with the optical PSF of the detection is performed using a PSF generated by the ImageJ plugin *PSF Generator*[3] with the Richard and Wolf model. Since the slight asymmetry that is created in a real OPM system is not taken into consideration, we use an optical PSF with an NA of 1.1 to not overestimate the optical performance of the system. The integrated intensity of the 3D PSF is also adjusted to account for the collection efficiency of the objective (~30% of all emitted light) and the estimated loss in the following optical detection path, which in the experimental setup was measured to be ~40-50%. To account for the oblique geometry of the detection, the volume generated by the 3D convolution is then rotated in 3D using a rotation angle corresponding to the angle of tilt of the tertiary objective. The final light intensity distribution falling on the detector is then extracted by taking the central slice of the rotated 3D intensity volume.

Detection

The intensity distribution given by the optical detection part is still associated with the emission spectrum assigned to the specific fluorophores. This allows for also incorporating the effect of emission filters to estimate more accurately the final intensity falling on the camera. For the simulation shown in Fig. 1d a band-pass filter transmitting 100% between 400-600 nm was used. To simulate the camera, the intensity extracted from the imaged volume is resampled onto a pixelated grid given the effective pixel size of the camera to be simulated which gives a final expected number of photons for each camera pixel. The expected number of photons is then converted to camera counts according to

$$C = o + \left(n_{ro} + \frac{Poiss(QE*E[p])}{K_{conv}}\right)$$

Where *C* is the final camera counts, *o* is the camera offset counts, n_{ro} is the read-out noise in e^- rms, *QE* is the quantum efficiency, *E[p]* is the expected number of photons falling on the pixel, K_{conv} is the photon conversion (electrons/count) factor and *Poiss(X)* denotes a Poisson distributed random number with expectation value *X*.

Supplementary Note 4: Optical build and alignment

The data presented in this publication were acquired using the custom microscope with an optical layout as depicted in Fig. 1e and detailed in the online methods section ‘Optical Setup’. The microscope is built on a vibrationally isolated optical table using standard optomechanical components. Below, the details of how the different parts of the setup were built and aligned are presented. The below sections primarily outline in detail the parts that are unique to our novel technology. For standard optical alignment procedures of sub-components shared with previous systems, we refer to previously published components and alignment protocols[4,5].

Primary objective and tube lens

The primary objective (O1) is mounted vertically with a 90-degree angled mirror mount placed underneath to reflect the vertical optical path to the horizontal table surface. The first tube lens (TL1) is then placed at the correct distance from the objective so that the two lenses form a 4f system and the alignment between the tube lens and objective is optimized. Next to the primary objective, the motorized stage for holding the sample is placed.

Galvanometric scanning unit

Next, the galvanometric scanning unit is placed before the tube lens. The scanning unit consists of a large galvanometric scanning mirror (GM) and two 2” mirrors placed in standard mirror holders. To align the scan unit, the incoming and outgoing optical axis are first defined using alignment lasers, one starting from above the mount of O1 and travelling in the ‘emission direction’ through the tube lens along the optical axis and one coming from the direction of the excitation lasers. In our design, we place the mirrors so that the output beam of the scanner leaves the assembly at a 90-degree angle from the input beam (Fig. 1e and Supplememtary Fig. 2a). The initial input and output axis thus needs to adhere to this. For later steps it is helpful to also place a small alignment plate (Thorlabs CPA1) in the path of the alignment laser coming from the objective side, placed so that the laser passes through the central hole. The first step of the scanner alignment is to place the galvanometric mirror (GM) where the two lasers intersect. This is done by moving the mirror around until both alignment lasers hit the mirror at the same spot. It is helpful to try to place the mirror at an angle as close as possible to the design angle of 25 degrees from the incoming excitation beam. After this, the other two mirrors are placed at positions and angles roughly corresponding to the positions and angles shown on the design sketches (Supplementary Fig. 2a). It is important to make sure that the gap between the mirrors is wide enough to allow the scanned laser beam to pass through without being cut. Keep only the laser from the excitation side (the one heading towards the objective) switched on for the rest of the procedure and try to make the beam path follow the intended path sketched in the design before fixing the mirrors to the table. Next, iteratively perform the following two steps, (i) move the fine adjusters on the mirror first hit by the alignment laser (M1) so that the laser beam hits the center of the galvanometric mirror also on the second reflection off it. (ii) move the fine adjusters on the second mirror (M2) so that the laser hits the previously placed alignment plate in the centre. When the laser hits the centre of the galvanometric mirror on the second reflection and the centre of the alignment plate, it is perfectly aligned with the predetermined output axis of the system and the scanning unit is aligned.

Oblique optical detection

The light emitted from the sample will pass through the primary objective, primary tube lens and scanning unit. After this, the secondary tube lens (TL2) will be placed and aligned using standard procedures to

form a 4f system with the primary tube lens. Throughout this procedure, it is helpful to use an alignment laser (preferably green) placed above the primary objective holder that travels through the system in the same way as the emission from the sample. After the secondary tube lens, the main dichroic mirror should be placed that transmits the emitted light from the sample but reflects the excitation lasers. After the main dichroic, the beam is reflected off two standard 1” mirrors before entering the secondary objective (Fig. 1e). The secondary objective is then aligned axially at the correct distance to form a 4f system with the secondary tube lens. The fine alignment of the beam to the objective is achieved through the fine adjusters of the mirror mounts placed before the secondary objective lens. It is important to note that if a secondary objective is used that is designed for imaging through a coverslip (such as is the case with the Nikon 40X 0.95 NA objective used in our setup), it is necessary to place such a coverslip between the secondary and tertiary objective to obtain optimal optical performance. The coverslip can either be glued onto the front surface of the objective (this service is offered by some retailers) or placed in some other way between the two objective lenses. The coverslip must be perfectly orthogonal to the optical axis to avoid aberrations.

The tertiary objective is mounted to have fine alignment motion available for both rotating the objective and translating it in all three spatial directions. With the alignment beam switched on and entering the secondary objective collimated, these fine adjusters can be used to position the objective so that the alignment beam exits the tertiary objective as a collimated beam aligned with the optical axis of the objective. When this is reached, the focal points of the secondary and tertiary objectives are superimposed. Care also needs to be taken to ensure that the objective is positioned at a 35-degree angle to the secondary objective. Once the tertiary objective is positioned correctly, the tertiary tube lens can be placed and aligned to the tertiary objective and the camera placed after the tube lens at the correct distance and position.

Illumination paths

The illumination paths consist of a widefield 405 nm illumination, a 488 nm interference pattern and a 488 nm light sheet illumination. Both the 488 nm illuminations (light sheet and interference pattern) need to be tilted 35 degrees to match the tilted detection. In practice, we first create and align the two 488 nm illuminations in a non-tilted configuration and then induce the tilt as the final step in alignment. Following this, the first step in creating the illuminations is to follow standard alignment procedures to configure the optical paths for the two 488 nm illuminations according to the design in Fig. 1e, but without the elements creating the bespoke patterns (the diffraction grating, physical mask for the diffraction orders and the cylindrical lens). The path that will be used for the interference pattern will then form a widefield illumination in the sample space and the path for the light sheet will form a focused illumination in the sample space.

The interference pattern is then created by placing the diffraction grating in the designated path and position outlined in the optical design and a physical mask after the following lens that lets only the +1 and -1 diffraction orders pass. This should now create an interference pattern in the sample space with the design periodicity. The light sheet illumination is created by placing the cylindrical lens in its designated place. The lens should be rotated to create a horizontal line directly after the lens. This illumination path should also contain two adjustable slits. The first one, placed before the cylindrical lens, should be adjustable in the vertical direction and is used to adjust the lateral width of the light sheet in sample space. The second one should be placed after the cylindrical lens and should be adjustable in the horizontal direction. This one is used to crop the line focused on the back focal plane limiting the focusing angle (effective NA), and thus adjusts the thickness, of the lights sheet.

Lastly, the widefield 405 nm illumination is coupled in through the shortpass dichroic mirror that reflects the 488 nm lasers and transmits the 405 nm laser as shown in Fig. 1e.

Alignment module for tilt and translation

The tilt and lateral position of the illuminations is adjusted using the two rotational platforms that compose the alignment module (AM) shown in Fig. 1e. On top of the two rotational platforms, two mirrors are places as shown in Supplementary Fig. 2b. When the platforms are rotated, the output beam undergoes a pure translation. Depending on whether the rotational platform is placed in the conjugate sample space or conjugate back focal plane space, this translation will correspond to either a translation or tilt in the sample space (Supplementary Fig. 2c). Within the alignment module (AM) the two rotational platforms are placed on either side of a lens, thus placing one in conjugate sample space and one in the conjugate back focal plane space. Rotating one of them (the first one along the illumination path) will cause a translation of the illumination patterns in the sample space while rotating the other will cause a tilt of the illumination patterns in the sample space. The two can thus be used together to achieve both the 35-degree tilt and to align the light sheet laterally to the detection plane of the tilted optical detection. The alignment of the patterns is visualized by performing a probing bead scan in the y-z plane and reconstructing an intensity image, as shown in Supplementary Fig. 3. To adjust to co-alignment between the off-pattern and the light-sheet pattern, we gently touch one of the mirrors in the part of the off-pattern path before the two paths are combined.

Supplementary Tables

Supplementary Table 1: Imaging parameters

Figure	Cell type	Plasmid/ Label	On-switching		Off-switching		488 nm Read-out			Volumetric FOV, x-y-z (µm)**	Acquisition time per volume (seconds)
			Peak intensity (W/cm^2)	Pulse length (ms)	Peak intensity (W/cm^2)	Pulse length (ms)	Peak intensity (W/cm^2)	Pulse length (ms)	Lat. Scan step		
2a	Virtual	rsEGFP2	80	15	50	40	500	1	105	-	-
Fig. 3 and Ext.Data. Fig. 1	HeLa	MAP2-rsEGFP(N205S)	75	20	50	200	410	1	105	90x42x11	10* (5)
Fig. 4, Ext.Data. Fig. 2, and Supp. Vid 1	HeLa	Actin-Chromobody-rsEGFP(N205S)	75	20	50	100	410	1	210	130x63x11	2
Fig. 5, and Supp. Vid. 2 and 3	HeLa	H2B-rsEGFP2	75	15	50	25	410	1	210	140x84x19	1.5
Fig. 6, Ext.Data. Fig. 3 and 4, and Supp.Vid 5	HeLa	Gag-IM-rsEGFP2	75	15	50	40	410	1	105	80x42x19	2
Supp.Vid. 4	HeLa	OMP25-rsEGFP2	75	15	50	25	410	0.5	210	57x42x8	0.7
Supp. Fig 5	HeLa	Gag-IM-rsEGFP2	75	15	50	40	410	1	105	74x42x14	2

* The data presented in Fig 2a and Extended Data. Fig. 1 is the average of two volumetric acquisitions, each acquired in 5 seconds.

** The given volumetric size corresponds to the data volume acquired. The image quality may vary within this volume due to limitations induced by optical system design choices and sample properties.

Supplementary Figures

Supplementary Figure 1

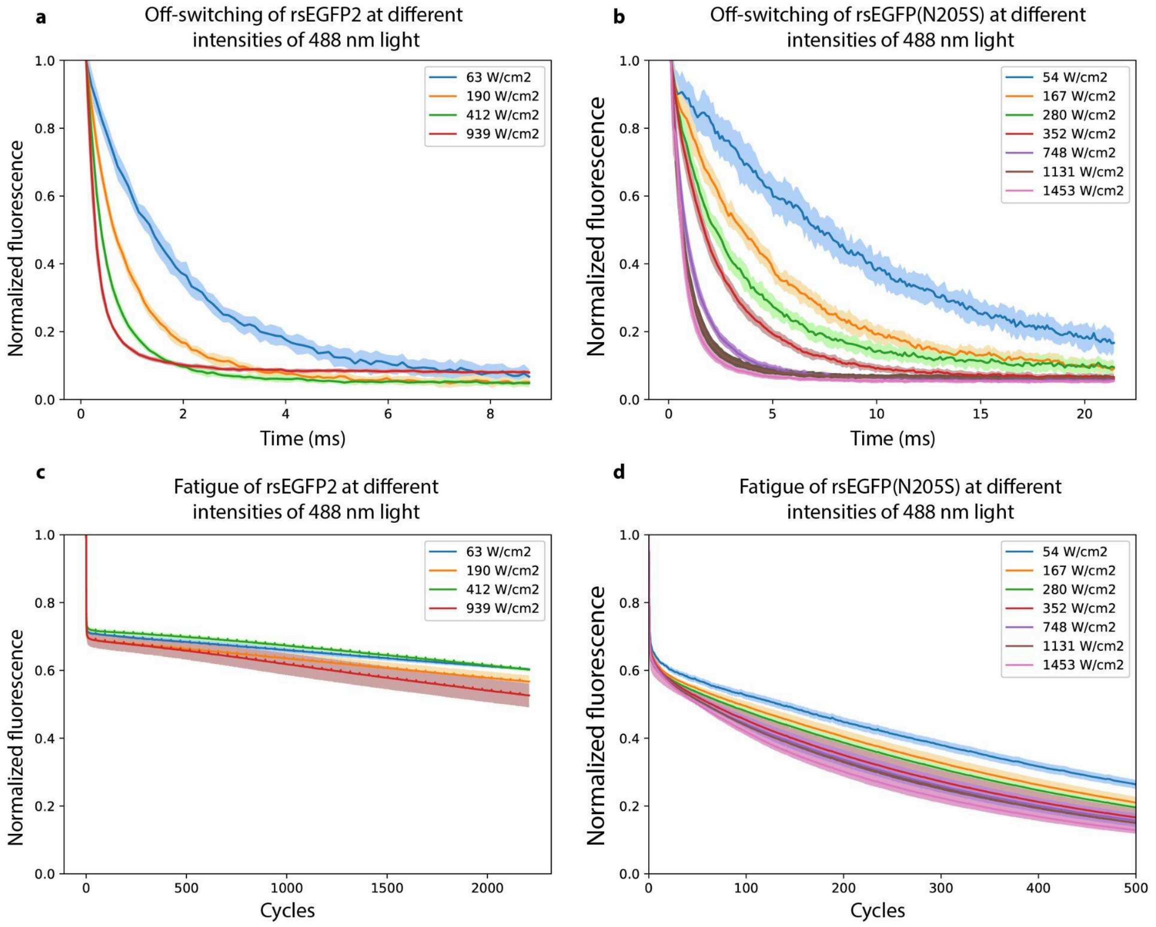

Supplementary Figure 1. RSFP off-switching and fatigue. The photoswitching rates and fatigue resistance are measured using purified proteins embedded in a polyacrylamide gel. **a)** Off-switching curves of rsEGFP2 under four different 488 nm illumination intensities. **b)** Off-switching curves of rsEGFP(N205S) under seven different 488 nm illumination intensities. All off-switching curves shown are the mean (sharp line) and ± one standard deviation (shaded regions) of 25-30 consecutive cycles. **c)** Fatigue curves of rsEGFP2 under four different 488 nm illumination intensities. **d)** Fatigue curves of rsEGFP(N205S) under seven different 488 nm illumination intensities. All fatigue curves shown are the mean (sharp line) and ± one standard deviation (shaded regions) of four repeated recordings.

Supplementary Figure 2

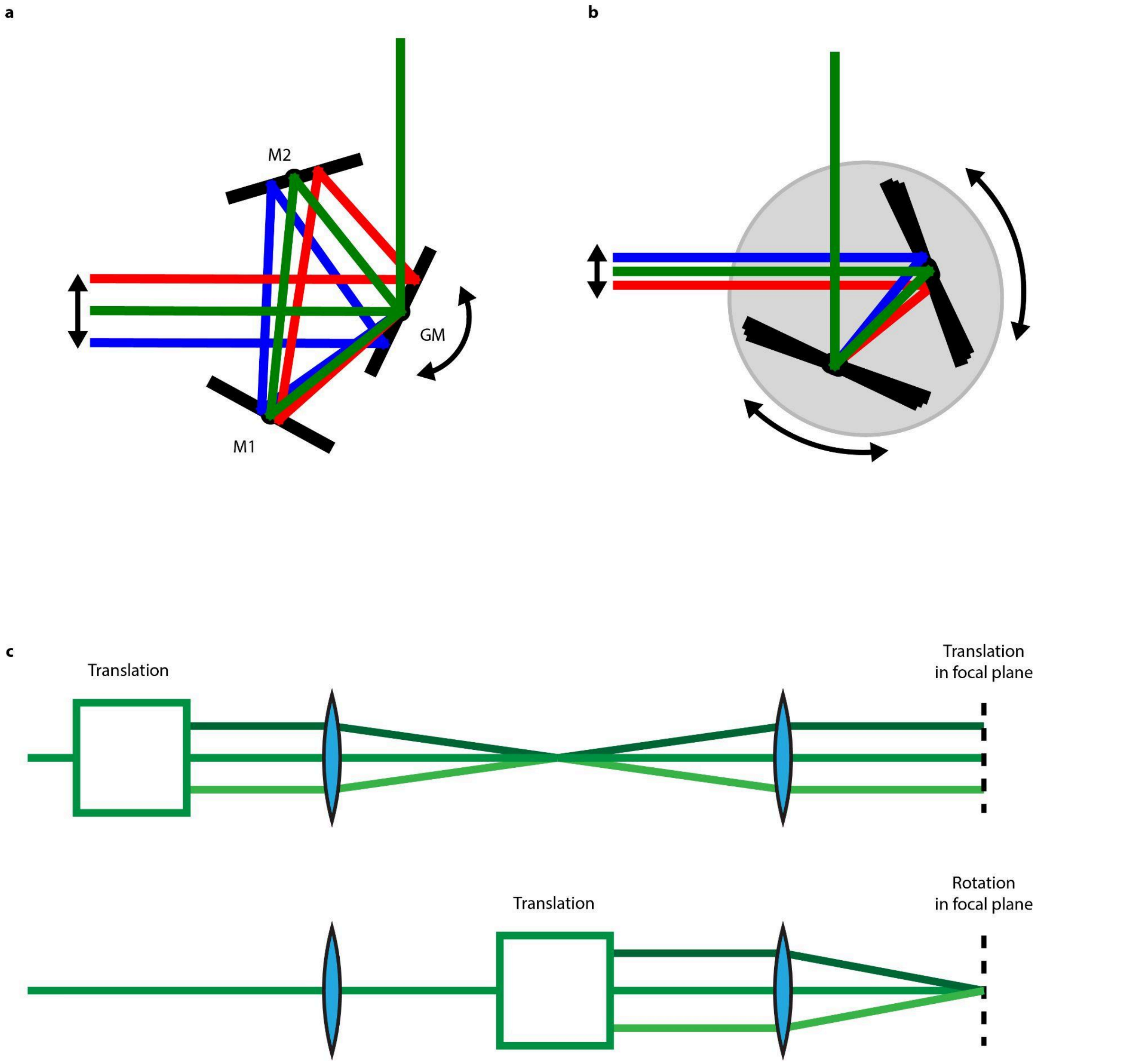

Supplementary Figure 2. Schematic images of scanning and alignment modules. a, Schematic image showing the beam path through the scanning module (SM in Fig. 1e). When the galvanometric mirror (GM) is rotated, an angular deflection is induced in the beam at the first reflection on GM. This angular deflection is then nullified at the second reflection on GM, leaving only a pure translation of the beam. Since only one mirror needs to be rotated, this assembly can be implemented for fast scanning due to the use of a galvanometric mirror. **b**, Schematic image showing the beam path through the beam translation assembly used in the alignment module (AM in Fig. 1e). Two mirrors are mounted on a rotation stage. Since the angle of the two mirrors are mechanically coupled the deflection angles will cancel out and result in a pure beam translation when the stage is rotated. The assembly is compact but since more mass needs to be moved it is better suited for slow movements e.g. alignment procedures. **c**, Schematic image showing how placing the translation module on different sides of a lens results in either a translation or angular rotation of the image in the focal plane of the optical system.

Supplementary Figure 3

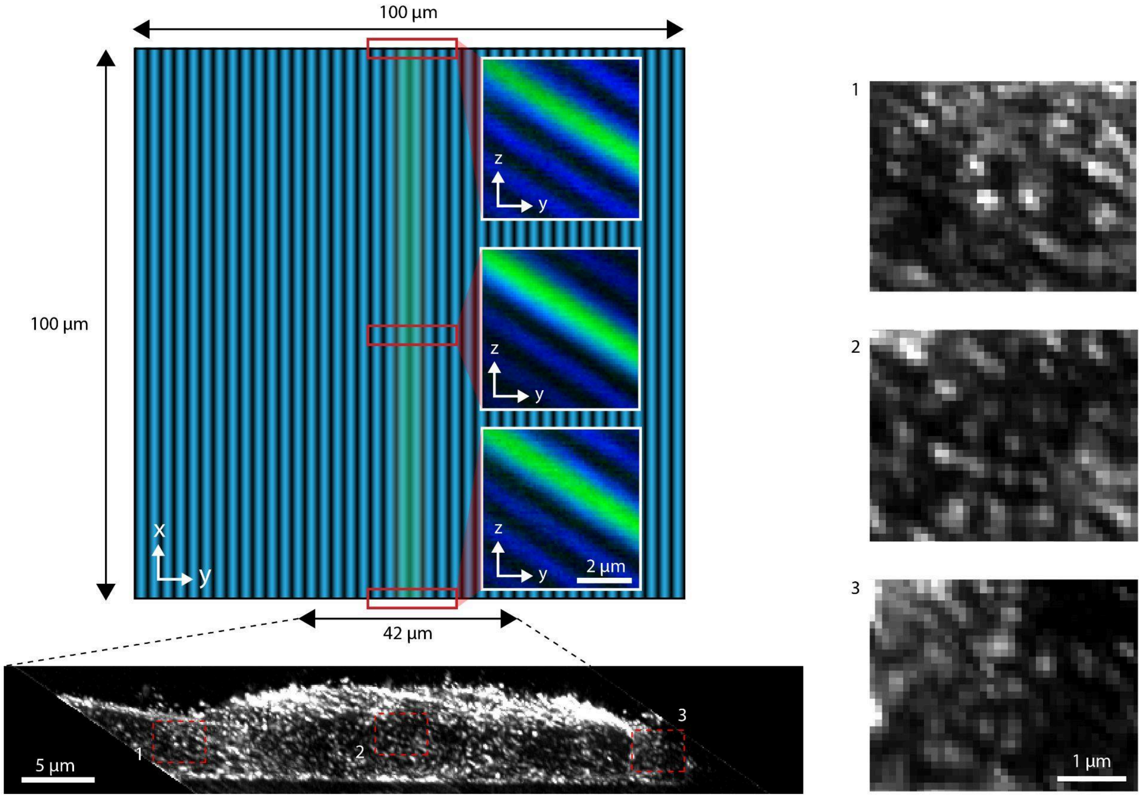

Supplementary Figure 3. Alignment over the FOV. The data shown in this panel demonstrates that the alignment of the illumination pattern, which is key to system performance, is maintained over the field of view imaged. The consistency of the alignment along the x-axis is checked by performing a bead scan to probe both the off and read-out pattern at three different positions along the x-axis. One bead scan is performed on each illumination pattern and position. The alignment along the y-axis is solely dependent on the consistent periodicity of the off pattern and the accurate calibration of the scanning step size during the read-out sweep. By investigating the image quality at three different positions along the y-axis, no apparent degradation of image quality is seen, indicating that the step size is well calibrated to the periodicity of the off-pattern. The data shown is a maximum intensity projection of the first time point of the time series presented in Fig 2c. The patterns shown inside the large x-y field of view are only schematic.

Supplementary Figure 4

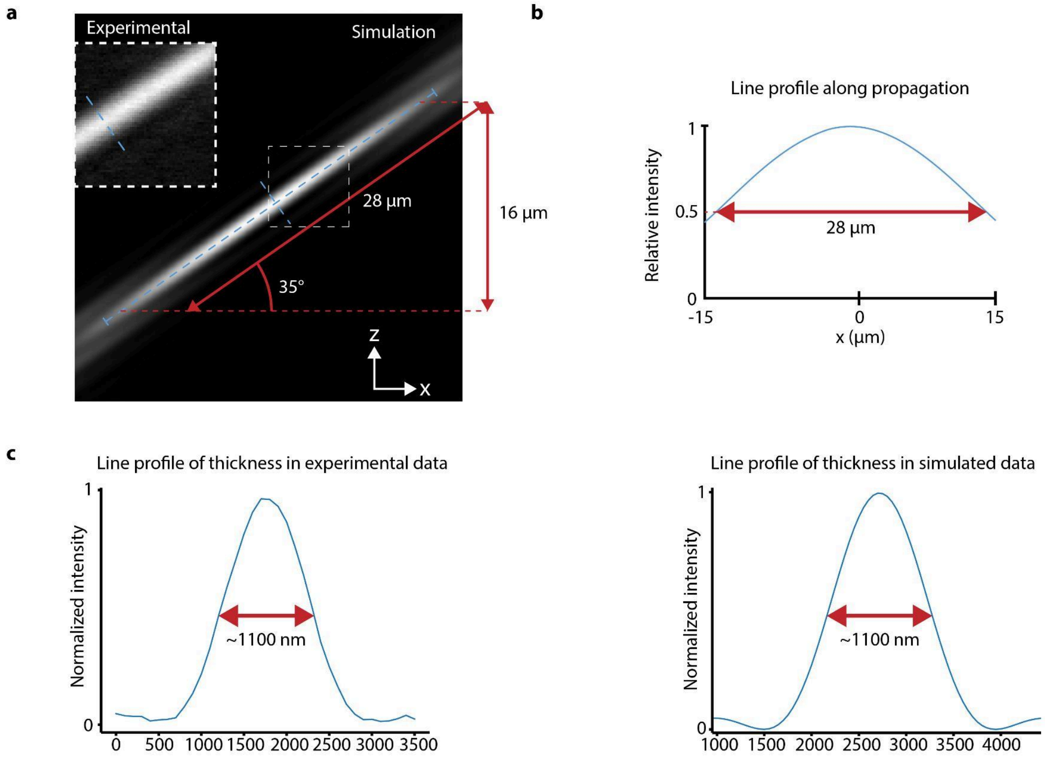

Supplementary Figure 4. Light-sheet propagation length. a) Simulation of the intensity distribution of the read-out light sheet used in the Multi-sheet RESOLFT recordings. **b)** Line profile of the intensity along the central lobe of the light sheet. The FWHM along the direction of propagation is 28 µm. This translated into a length along the z-axis of 16 µm. **c)** Line profile drawn and measured perpendicular to the direction of propagation of the light sheet in both an experimentally measured intensity profile (left graph) and in the simulated intensity profile (right graph). The experimental data is the same as shown in the middle of Supplementary Fig. 3. Both graphs show a FWHM thickness of close to 1100 nm.

Supplementary Figure 5

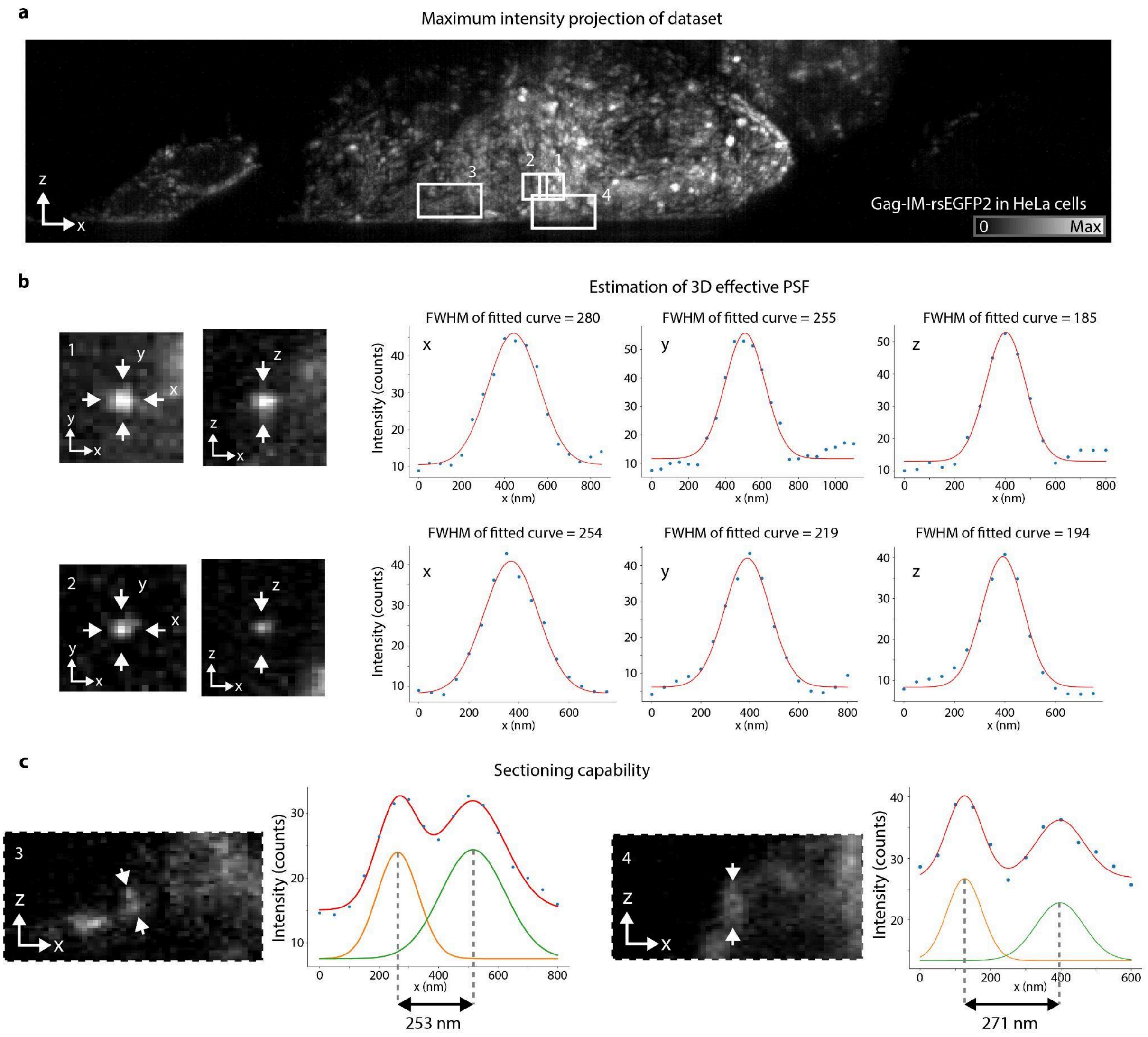

Supplementary Figure 5. Quantification of resolution. To quantify the achievable resolution in the Multi-sheet RESOLFT system, we reconstructed a volumetric image of a HeLa cell transfected with Gag-IM-rsEGFP2 from an acquisition consisting of 5 consecutive full volumetric recordings. This creates a reconstruction with minimal spurious noise, allowing for a more accurate quantification of x-y-z resolution and sectioning capability. All data shown is reconstructed with the simple deskew algorithm i.e. not using any deconvolution.. **a)** A maximum intensity projection of the reconstructed volume along the y-direction shows the outline of the sample and the clear clustering tendency of the Gag proteins. **b)** By selecting two small and isolated protein clusters (location indicated by red rectangles in panel **a**) the effective PSF size can be estimated along the x, y and z directions. The sizes in the x and y directions are determined by the optical resolution of the system (~250 nm) and the size along the z-direction is determined by the active confinement from the off-switching illumination pattern. The size of ~190 nm along z corresponds to maximum confinement along the 35-degree tilted direction of ~150-160 nm. **c)** The enhanced sectioning capability of the system is further demonstrated by identifying two structures in the sample where axially separated structures are distinguishable at a distance just above the axial size of the effective PSF.

References

1. Bourges, A. C. *et al.* Quantitative determination of the full switching cycle of photochromic fluorescent proteins. *Chem. Commun.* **59**, 8810–8813 (2023).
2. Bodén, A. *et al.* Volumetric live cell imaging with three-dimensional parallelized RESOLFT microscopy. *Nat. Biotechnol.* **39**, 609–618 (2021).
3. Hagai Kirshner, Daniel Sage. PSF Generator.
4. Sapoznik, E. *et al.* A versatile oblique plane microscope for large-scale and high-resolution imaging of subcellular dynamics. *eLife* **9**, e57681 (2020).
5. Andrew G York, Alfred Millett-Sikking. High NA single-objective light-sheet. Preprint at https://doi.org/doi:10.5281/zenodo.3244420 (2019).
