# Supplementary material for: Super-sectioning with multi-sheet reversible saturable optical fluorescence transitions (RESOLFT) microscopy
Source: Nat Methods. 2024 Feb 23;21(5):882–8. doi: 10.1038/s41592-024-02196-8 (PMC11093742; doi:10.1038/s41592-024-02196-8)
Supplement: Supplementary file 2 — Reporting Summary [file 41592_2024_2196_MOESM2_ESM.pdf]

nature portfolio

Corresponding author(s): Ilaria Testa

Last updated by author(s): Nov 12, 2023

Reporting Summary

Nature Portfolio wishes to improve the reproducibility of the work that we publish. This form provides structure for consistency and transparency in reporting. For further information on Nature Portfolio policies, see our Editorial Policies and the Editorial Policy Checklist.

Statistics

For all statistical analyses, confirm that the following items are present in the figure legend, table legend, main text, or Methods section.

n/a	Confirmed	
☐	☒	The exact sample size (*n*) for each experimental group/condition, given as a discrete number and unit of measurement
☐	☒	A statement on whether measurements were taken from distinct samples or whether the same sample was measured repeatedly
☒	☐	The statistical test(s) used AND whether they are one- or two-sided<br>*Only common tests should be described solely by name; describe more complex techniques in the Methods section.*
☒	☐	A description of all covariates tested
☒	☐	A description of any assumptions or corrections, such as tests of normality and adjustment for multiple comparisons
☐	☒	A full description of the statistical parameters including central tendency (e.g. means) or other basic estimates (e.g. regression coefficient) AND variation (e.g. standard deviation) or associated estimates of uncertainty (e.g. confidence intervals)
☒	☐	For null hypothesis testing, the test statistic (e.g. *F*, *t*, *r*) with confidence intervals, effect sizes, degrees of freedom and *P* value noted<br>*Give P values as exact values whenever suitable.*
☐	☒	For Bayesian analysis, information on the choice of priors and Markov chain Monte Carlo settings
☒	☐	For hierarchical and complex designs, identification of the appropriate level for tests and full reporting of outcomes
☒	☐	Estimates of effect sizes (e.g. Cohen's *d*, Pearson's *r*), indicating how they were calculated

Our web collection on statistics for biologists contains articles on many of the points above.

Software and code

Policy information about availability of computer code

Data collection	The data was acquires using a hardware control software based heavily on the ImSwitch architechture. This exact software version used, together with the firmware used on the Triggerscope 4.0 can be found on https://github.com/TestaLab/Multi-sheet-RESOLFT_code. Dependencies and version numbers are listed in each repository.
Data analysis	The image reconstruction was done using a custom written reconstruction and deconvolution software that is available at https://github.com/TestaLab/Multi-sheet-RESOLFT_code. Dependencies and version numbers are listed in each repository. The source code for the software used for the simulations is currently not publicly accessible, but its working principles are described in detail in Supplementary Note 3.

For manuscripts utilizing custom algorithms or software that are central to the research but not yet described in published literature, software must be made available to editors and reviewers. We strongly encourage code deposition in a community repository (e.g. GitHub). See the Nature Portfolio guidelines for submitting code & software for further information.

Data

Policy information about availability of data

All manuscripts must include a data availability statement. This statement should provide the following information, where applicable:

- Accession codes, unique identifiers, or web links for publicly available datasets
- A description of any restrictions on data availability
- For clinical datasets or third party data, please ensure that the statement adheres to our policy

All the data presented is available as both raw dataset and reconstructed volumes at Zenodo https://doi.org/10.5281/zenodo.10474256. Due to space limitations, all the reconstructed volumes could not be published, but are available from the authors upon reasonable request.

Research involving human participants, their data, or biological material

Policy information about studies with human participants or human data. See also policy information about sex, gender (identity/presentation), and sexual orientation and race, ethnicity and racism.

Reporting on sex and gender	N/A
Reporting on race, ethnicity, or other socially relevant groupings	N/A
Population characteristics	N/A
Recruitment	N/A
Ethics oversight	N/A

Note that full information on the approval of the study protocol must also be provided in the manuscript.

Field-specific reporting

Please select the one below that is the best fit for your research. If you are not sure, read the appropriate sections before making your selection.

☒ Life sciences ☐ Behavioural & social sciences ☐ Ecological, evolutionary & environmental sciences

For a reference copy of the document with all sections, see nature.com/documents/nr-reporting-summary-flat.pdf

Life sciences study design

All studies must disclose on these points even when the disclosure is negative.

Sample size	The study does not present any data based on statistical analysis and no sample sizes were therefore chosen. Images presented are on individual samples and line-profiles measured are on individual filaments or puncta structures. Imaging performance is demonstrated in multiple different sample types (N = 3) imaged on different days in order to demonstrate the techniques versatility.
Data exclusions	No data were excluded from the analysis.
Replication	After system alignment recordings could be performed over the coarse of a full day without notable deterioration in image quality. After closing down the system and starting up the following day or multiple days after, minor realignments were sometimes needed. Imaging was performed inside a time period of half a year. All attempts at replication were successful.
Randomization	No allocation into experimental group were performed.
Blinding	No allocation into experimental group were performed.

Reporting for specific materials, systems and methods

We require information from authors about some types of materials, experimental systems and methods used in many studies. Here, indicate whether each material, system or method listed is relevant to your study. If you are not sure if a list item applies to your research, read the appropriate section before selecting a response.

Materials & experimental systems

n/a	Involved in the study
☒	☐ Antibodies
☐	☒ Eukaryotic cell lines
☒	☐ Palaeontology and archaeology
☒	☐ Animals and other organisms
☒	☐ Clinical data
☒	☐ Dual use research of concern
☒	☐ Plants

Methods

n/a	Involved in the study
☒	☐ ChIP-seq
☒	☐ Flow cytometry
☒	☐ MRI-based neuroimaging

Eukaryotic cell lines

Policy information about cell lines and Sex and Gender in Research

Cell line source(s)	Hela, ATCC CCL-2
Authentication	None of the cell lines used were authenticated.
Mycoplasma contamination	No testing for mycoplasma contamination.
Commonly misidentified lines (See ICLAC register)	No commonly misidentified cell lines were used in this study.
